# Health workforce skill mix and task shifting in low income countries: a review of recent evidence

**DOI:** 10.1186/1478-4491-9-1

**Published:** 2011-01-11

**Authors:** Brent D Fulton, Richard M Scheffler, Susan P Sparkes, Erica Yoonkyung Auh, Marko Vujicic, Agnes Soucat

**Affiliations:** 1 Global Center for Health Economics and Policy Research, School of Public Health, University of California-Berkeley, Berkeley, USA; 2 School of Public Health, Harvard University, Cambridge, USA; 3 Graduate School of Social Welfare, Ewha Womans University, Seoul, Korea; 4 Human Development Network, The World Bank, Washington DC, USA; 5 Human Development, African Development Bank, Tunis-Belvedère, Tunisia

## Abstract

**Background:**

Health workforce needs-based shortages and skill mix imbalances are significant health workforce challenges. Task shifting, defined as delegating tasks to existing or new cadres with either less training or narrowly tailored training, is a potential strategy to address these challenges. This study uses an economics perspective to review the skill mix literature to determine its strength of the evidence, identify gaps in the evidence, and to propose a research agenda.

**Methods:**

Studies primarily from low-income countries published between 2006 and September 2010 were found using Google Scholar and PubMed. Keywords included terms such as skill mix, task shifting, assistant medical officer, assistant clinical officer, assistant nurse, assistant pharmacist, and community health worker. Thirty-one studies were selected to analyze, based on the strength of evidence.

**Results:**

First, the studies provide substantial evidence that task shifting is an important policy option to help alleviate workforce shortages and skill mix imbalances. For example, in Mozambique, surgically trained assistant medical officers, who were the key providers in district hospitals, produced similar patient outcomes at a significantly lower cost as compared to physician obstetricians and gynaecologists. Second, although task shifting is promising, it can present its own challenges. For example, a study analyzing task shifting in HIV/AIDS in sub-Saharan Africa noted quality and safety concerns, professional and institutional resistance, and the need to sustain motivation and performance. Third, most task shifting studies compare the results of the new cadre with the traditional cadre. Studies also need to compare the new cadre's results to the results from the care that would have been provided--if any care at all--had task shifting not occurred.

**Conclusions:**

Task shifting is a promising policy option to increase the productive efficiency of the delivery of health care services, increasing the number of services provided at a given quality and cost. Future studies should examine the development of new professional cadres that evolve with technology and country-specific labour markets. To strengthen the evidence, skill mix changes need to be evaluated with a rigorous research design to estimate the effect on patient health outcomes, quality of care, and costs.

## Introduction

In *Working Together for Health: The World Health Report 2006*, WHO estimated that countries that had fewer than 2.28 doctors, nurses, and midwives per 1000 population were, on average, unable to achieve an 80% coverage rate for deliveries by a skilled birth attendant [1]. WHO found that 57 countries fall short of that threshold, resulting in a needs-based shortage of 4.3 million health workers, including 2.4 million doctors, nurses, and midwives. In addition to the workforce shortage, the report emphasizes three other workforce challenges: skill mix imbalances, urban-rural distribution imbalances, and poor working conditions, including compensation. With regard to skill mix, the report states: "In many countries, the skills of limited yet expensive professionals are not well matched to the local profile of health needs" (p. xviii). When the skill mix and each cadre's activities and tasks are not well matched to the local health care need, then health care services become less accessible, and even when they are accessible, they become less affordable.

This article provides a review of the health workforce skill mix literature, focusing on task shifting in low-income countries. Task shifting is defined as delegating tasks to existing or new cadres with either less training or narrowly tailored training. Dovlo describes various task shifting scenarios, such as shifting tasks from higher- to lower-skilled health workers (e.g. from a nurse to a community health worker) [2]. Task shifting also includes the creation of new professional or non-professional cadres, whereby tasks are shifted from workers with more general training to workers with specific training for a particular task (e.g. assistant medical officers trained in obstetrics in Mozambique).

The primary objective of task shifting is to increase productive efficiency, that is, to increase the number of health care services provided at a given quality and cost, or, alternatively, to provide the same level of health care services at a given quality at a lower cost. The efficiency gain from changing the skill mix of health workers could result in a number of improvements, such as increased patient access, a reduction in health worker training and wage bill costs, and a reduction in the health workforce needs-based shortage. Another objective of task shifting is to reduce the time needed to scale up the health workforce, because the cadres performing the shifted tasks require less training. While task shifting has been occurring for decades, it is seen by some as becoming more urgent, because of health care needs for HIV/AIDS patients and overall health worker needs-based shortages [3].

This article uses an economics perspective to examine the strength of the evidence on task shifting, to identify gaps in the evidence, and to propose a research agenda. The article is organized as follows: the introductory section continues by describing an economic-based conceptual framework to analyze skill mix policies; the second section describes the methods and data used to select studies to include in the literature review; section three summarizes the studies' results; and section four proposes a research agenda. Additional file 1 is appended as the final section, which includes a table that summarizes the important elements of each study that was included.

### Economic framework to evaluate skill mix

The skill mix of health workers within a health workforce significantly impacts the delivery of health care services. At a given facility, the optimal skill mix is the combination of health workers that produce a given level of health care services at a particular quality for the lowest cost. In economic terms, this mix of workers is defined as 'productively efficient'.

Palmer and Torgerson distinguish among technical efficiency, productive efficiency, and allocative efficiency [4]. Technical efficiency refers to the relationship between inputs and outputs, whereby a technically efficient relationship produces the maximum output, given the inputs. Productive efficiency extends technical efficiency to incorporate input costs. Productive efficiency is achieved when the maximum output is produced with a given budget for inputs, or alternatively, it is achieved when a given level of output is produced with the least costly mix of inputs. Productive efficiency implies technical efficiency, although the converse is not necessarily true. Allocative efficiency extends productive efficiency to incorporate the output's value to society. Allocative efficiency is achieved when economic social welfare is maximized, which occurs when the marginal social benefit of the output (i.e. its price, under free market conditions) equals the marginal social cost to produce the output. Allocative efficiency implies productive efficiency, although the converse is not necessarily true. Note that allocative efficiency does not consider equity.

**Figure 1:**
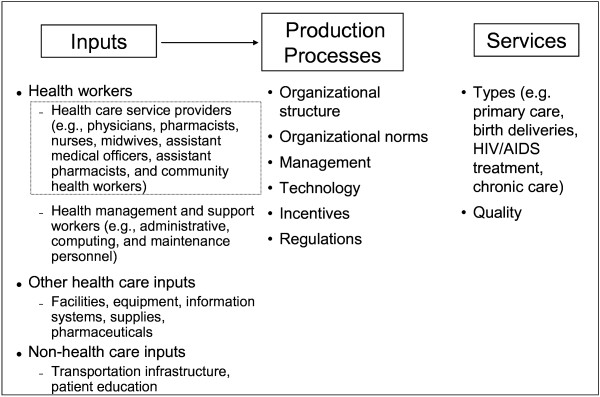
**Health Care Services Production Process**.

Figure 1 provides a stylized health care production process to illustrate the factors that influence the productively efficient mix of workers. This optimal mix of health workers is influenced by (1) the other health care inputs that are used; (2) the production processes that utilize the inputs to create health care services; and (3) the type and quality of services that are produced. The types of health workers include both health care service providers (e.g. physicians, pharmacists, nurses, midwifes, assistant medical officers, assistant pharmacists, and community health workers [see dotted interior box]) and health management and support workers (e.g. administrative, computing, and maintenance personnel). Other health care inputs include facilities, equipment, information systems, supplies, and pharmaceuticals, as well as non-health care inputs such as transportation infrastructure and patients' education levels. The production processes use these inputs to produce health care services, and the processes are affected by organizational structure, organizational norms, management, technology, incentives, and regulations. The type of service provided (e.g. primary care, birth deliveries, HIV/AIDS antiretroviral therapy, chronic care) and its level of quality will also influence which mix of workers is productively efficient. Because the above factors vary within and across countries, the external validity of many of the studies is relatively weak because the productively efficient skill mix depends on these local factors.

**Figure 2:**
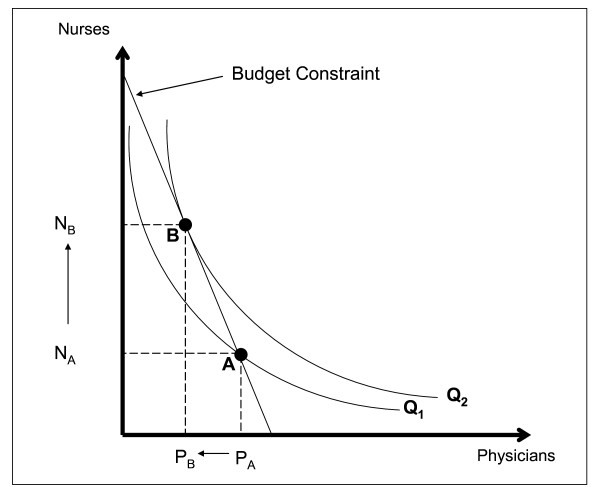
**Productively Efficient and Inefficient Skill Mixes**. This figure was based on well-known figures illustrating productive efficiency in economic textbooks e.g. [67].

There are many combinations of health worker skill mixes that could produce a health care service in a particular setting. Figure 2 illustrates the lowest-cost skill mix that can be used to produce a particular quantity of a given health care service at a given level of quality. It assumes a scenario in which two health worker types are available, physicians and nurses, but the same approach could be used to determine the productively efficient number of other health workforce cadres as well as non-human resource inputs for various health care services. In the figure, the horizontal axis represents the number of physicians, and the vertical axis represents the number of nurses. The straight line that intersects each axis represents a fixed budget constraint along which total staffing costs are equal. The budget constraint intersects the horizontal axis where the entire budget is used for physicians (i.e. the number of physicians will be the total budget divided by the physician wage); and the budget constraint intersects the vertical axis where the entire budget is used for nurses (i.e. the number of nurses will be the total budget divided by the nurse wage). The budget constraint could incorporate amortized training costs. The curved line Q_1 _is an isoquant that represents a particular quantity of the health care service that is produced by different mixes of physicians and nurses. The second curved line Q_2 _represents another particular quantity that is greater than Q_1_. The figure shows a productively inefficient skill mix (Point A) and a productively efficient skill mix (Point B). Point A is not productively efficient because the service provider could decrease the number of physicians from P_A _to P_B _and simultaneously increase the number of nurses from N_A _to N_B_. This skill-mix change would not increase costs, but would produce a higher quantity of health care services (Q_2 _> Q_1_). The productively efficient mix of workers is the point where the budget constraint is tangent to the isoquant, where the quantity of services at a given quality is maximized, subject to the available budget. Alternatively, the productively efficient mix can be thought of as the mix for which a given quantity of services at a particular quality is produced for the lowest cost.

Studies point to evidence that countries may not be operating at the productively efficient mix. For example, in 2003, the ratio of nurses to doctors was 8 to 1 in Africa and 1.5 to 1 in Western Pacific countries [1]. Hongoro and McPake show low- and middle-income countries that have a physician-to-nurse ratio greater than the global average (0.43), including Brazil (4.04), Bangladesh (0.96), and India (0.83) [5]. Zurn et al. show skill-mix variation within countries with similar economic development, and Gupta et al. show skill-mix variation within and between developed and transitional-economy countries [6,7]. Even with the difficulties in comparing cadre definitions across countries with different health care systems, such variations clearly suggest that countries are operating at different efficiency levels in terms of skill mix. However, the productively efficient skill mix will vary across and within countries, because of the different health care services being provided and because of different contextual factors, such as the health system, payment scheme, workforce training, and management culture.

If the skill mix is not at the productively efficient point, the potential inefficiencies are significant. For example, Fulton and Scheffler examined 84 low- and middle-income non-African countries, and estimated that 12 countries would experience a needs-based shortage of doctors, nurses, and midwives in 2015, totalling 581 000 health care professionals, costing $1.8 billion (2007 U.S. dollars) per year to eliminate [8]. Based on simulations, they estimated the percent reduction in the additional wage bill resources required to fill these shortages under three different scenarios of substituting community health workers (CHW) for nurses and midwives.

All three scenarios increased the needed number of nurses and midwives relative to doctors. In the first, or baseline, scenario, no nurses and midwives were replaced with CHWs. In the second and third scenarios, 10% and 20%, respectively, of each country's needed nurses and midwives were replaced with CHWs. For each scenario, the number of doctor equivalents was the same, whereby nurses, midwives, and CHWs were converted into doctor-equivalents. A nurse's or midwife's productivity was assumed to equal 0.8 of a doctor's, based on estimates in the United States, because there are few reliable estimates of this relative productivity factor in low- and middle-income countries [9-11]. A CHW's productivity was assumed to equal 0.3 of a nurse's or midwife's, and a CHW's wage was assumed to be 0.2 of a nurse's or midwife's. Because of the lack of CHW studies estimating productivity and wages, the relative CHW productivity and wage as compared to a nurse or midwife were based on the authors' preliminary assessment, and the authors realize these estimates will vary across countries. The relative productivity factor could be estimated at a facility level using time and motion studies (e.g. see Kurowski et al. [12]). When the needed nurse-plus-midwife-to-doctor ratio was increased by 50% in each of the 12 countries, the overall reduction in the annual wage bill shortage was 4%. Under that new ratio, when 10% of the needed nurses and midwives were replaced with CHWs, the annual wage bill reduction grows to 10%; when 20% of the needed nurses and midwives were replaced, the annual wage bill reduction grows to 15%.

Economic factors will not be the only influence governing skill mix decisions. Health care worker associations and licensure requirements define workers' scope of practice and can influence the extent to which the ratio of, for example, doctors to nurses can be altered [9].

If sufficient data exist, the facility or firm-level studies can be aggregated up to the country level to determine the productively efficient skill mix for a country. This type of aggregation is important, as the determination of the optimal mix of health worker cadres has important implications on country-level budgetary planning and training.

## Methods and data

We examined different methods to conduct our literature review. A systematic literature review is a common method, but it is better suited for a narrowly defined research question [13,14]. Because our research scope was broad, we followed the steps below to review the literature. These steps were based on the guidelines for a systematic literature review by the Centre for Reviews and Dissemination and adjusted for our article:

1. Determine research areas

2. Determine eligibility criteria for study selection

- search Google scholar using keywords

- limit studies to primarily include low-income countries

- limit time range to primarily between 2006 and September 2010

- select studies based on strength of evidence (i.e. research design, methods, and statistical significance of results)

3. Conduct search based on the above eligibility criteria to select studies

4. Evaluate studies, primarily based on research design, methods, and health care topic

5. Extract key information from selected studies, such as research design, methods, and results

6. Summarize results with suggestions for future research

Steps 1, 2, and 5 are discussed in further detail next. The research area included skill mix, with an emphasis on task shifting among health care service providers in low-income countries. The skill-mix studies examined health outcomes, health care utilization, and budget impacts of different skill mixes of workers.

We searched for studies on skill mix using Google Scholar with the following keywords: skill mix, task shifting, assistant medical officer, assistant clinical officer, assistant nurse, auxiliary nurse, enrolled nurse, auxiliary health worker, health care assistant, assistant pharmacist, and community health worker, as well as various combinations of these keywords. Google Scholar's ranking system heavily weights an article's citation count [15]. We supplemented the Google Scholar search using PubMed to search for additional select articles. We obtained additional studies from the authors' knowledge of relevant studies as well as examining the bibliographies of recent studies. We selected 31 studies to critically analyze, based on the strength of evidence presented (i.e. research design, methods, and statistical significance of results) and how recently they were published. We mostly searched for studies published between 2006 and September 2010, but included earlier studies when there was a compelling reason (e.g. high strength of evidence).

The elements we used to describe the studies included the following: research question(s), population studied, study design, analytic method, and key results. These elements are presented for each of the 31 studies in a table (see Additional file 1). The research question(s) included the study's primary research questions, whether a health workforce intervention was tested, and related policy questions.

The population studied was defined along several dimensions, including the geographical location, year(s), unit of analysis (e.g. patient, health worker, health facility); data source (e.g. survey, administrative records, or a trade association); data structure (e.g. cross-section, repeated cross-section, and longitudinal); and sample size.

There were seven study designs, ordered by the strength of evidence: randomized controlled trial (known as an experimental design), quasi-experimental, multi-group comparison, forecast, case study, descriptive study, and literature review. A study was considered to be a randomized controlled trial if treatments (e.g. skill mixes) were randomly assigned to patients. Quasi-experimental studies included those for which the skill mix assignment was the result of an exogenous policy that was not directly related to the outcome of interest (e.g. patient outcomes; see Barber et al. [16]). Multi-group comparison studies included those for which two or more groups of workforce cadres were compared to each other, based on measures such as patient outcomes or costs; however, the patients were not randomly assigned to the workforce cadre, so the potential for confounding factors biasing the estimated results is high. Forecast studies included those for which forecasts were prominent. A study was considered to be a case study if it used formal case study protocols [17]. A study was considered descriptive if it did not use formal protocols, and relied primarily on qualitative assessment rather than quantitative evidence. The descriptive studies usually examined a specific health workforce issue, and in many cases argued for a particular viewpoint based on the author(s)' expertise and judgment. We included literature reviews as part of our review, but primarily relied on original research.

The two types of analytic methods were quantitative and qualitative. A quantitative method was denoted when data analysis strongly influenced the findings. A qualitative method, typically used for a descriptive evaluation, was denoted when the author's/authors' findings were based on key-informant interviews and their own expertise and judgment. When quantitative methods were used, we noted whether the method involved descriptive statistics, comparing means, or multivariate regression analysis. For a literature review, the analytic methods included systematic review (e.g. meta-analysis), structured review (i.e. protocols for study selection were documented), and unstructured review (i.e. protocols for study selection were not documented).

## Results

Many of the health workforce skill mix studies examined whether patient health outcomes, quality of care, and costs differed among different skill mixes of health care service providers. The studies examined task shifting, particularly the development of new professional cadres designed to increase productive efficiency and reduce the time needed to scale up, resulting in increased patient access and a reduction in health worker training and wage bill costs.

Task shifting includes various scenarios, such as substituting tasks among professionals, delegating tasks to professionals with less training, including creating a new cadre, delegating tasks to non-professionals, or a combination of these [2]. For example, the work can shift from specialist physicians to general practitioners, nurses, midwives, or assistant medical officers. Other cadre titles that participate in task shifting include clinical officer, assistant clinical officer, assistant nurse, auxiliary nurse, enrolled nurse, auxiliary health worker, health care assistant, assistant pharmacist, and community health worker.

The work can also be redistributed according to new categories of health workers. There are many examples of new professional cadres being developed, from health extension workers being trained in one year in vocational schools in Ethiopia, to assistant medical officers being trained in obstetrics in Mozambique, to physician assistants being trained in the United States [18-20]. Task shifting, including the development of new professional cadres, has been occurring for decades in both high-income countries (e.g. in the USA, see Hooker) and low-income countries, but is seen by some as becoming more urgent in low-income countries because of health care needs for HIV/AIDS patients and overall health worker needs-based shortages [3,20,21].

The review produced three main findings. First, the studies provide substantial evidence that task shifting is an important policy option to help alleviate health workforce shortages and skill mix imbalances, whether the shortages and imbalances are needs-based or economic demand-based. This finding is supported by other recent reviews of task shifting, including HIV/AIDS treatment and care provided by lay and community health workers in Africa, maternal and child health care as well as the management of infectious diseases by lay health workers, and doctor-nurse substitution in primary care in developed countries [22-24]. As we discuss below, the reviews emphasized the success of task shifting depends on local contextual factors. Although the studies that evaluated task shifting were typically not based on an experimental design such as a randomized controlled trial (as noted by, e.g. Buchan and Dal Poz; and by Zurn et al.), there is substantial evidence from non-experimental studies [6,25].

Several example studies are discussed next, and the first two are based on randomized controlled trials. In Kenya, no significant clinical differences were found between HIV/AIDS patients who received clinic-based antiretroviral therapy care versus primarily community-based care delivered by people living with HIV/AIDS who received pre-programmed personal digital assistants with decision support [26]. In Uganda, non-physician clinicians (NPC) and physicians had considerable strength of agreement for HIV/AIDS patient assessment, particularly with the final antiretroviral therapy (ART) recommendation, WHO clinical stage assignment, and tuberculosis status assessment [27]. Surgically trained assistant medical officers (*tecnicos de cirurgia *[TC]) in Mozambique produced similar patient outcomes as compared to physician obstetricians and gynecologists, but the TC's cost of surgery was estimated to be one-quarter of physician specialists, and TC's provided over 90% of obstetric surgery delivered in district hospitals [19,28]. Clinical officers and medical officers providing obstetric surgery in Malawi produced similar patient outcomes [29]. Huicho and colleagues found that the number of years of pre-service training was generally not associated with the appropriate assessment, diagnosis, and treatment of young children in Bangladesh, Brazil, Tanzania, and Uganda [30]. Lekoubou and colleagues reviewed the evidence of nurses managing chronic conditions, specifically hypertension and diabetes mellitus in sub-Saharan Africa, and concluded that they are a potentially promising cadre to efficiently manage these chronic conditions [31]. While nurse-led care is common in sub-Saharan Africa, nurse-led care with a specific application to chronic diseases is relatively new.

In a mental health example, which used an experimental design, Rahman and colleagues found that lady health workers (community health workers) in Pakistan trained in cognitive behaviour techniques significantly lowered depression prevalence among new mothers more than lady health workers without the training [32]. While outcomes were not compared to physician specialists and other psychosocial care providers, the study demonstrates the potential to train CHWs in mental health treatments (also see Patel [33]). This is important, given that there is a large needs-based shortage of mental health workers in low- and middle-income countries [34,35].

Second, while there is substantial evidence that task shifting has the potential to increase productive efficiency and reduce the time needed to scale up, there are a number of challenges, and results have not always been favourable. In the study by Zachariah et al. of task shifting in HIV/AIDS in sub-Saharan Africa, they note quality and safety concerns, professional and institutional resistance, and the need to sustain motivation and performance [36]. For example, quality of care may decrease if CHWs are given complex tasks. In Kenya, where CHWs had broad responsibilities of diagnosing and treating children, a study found that 80% of all guideline-recommended procedures were performed correctly, but only 58% of ill children were prescribed all potentially life-saving treatments [37]. The same is true in high-income countries: Buchan and Calman found that many questions remain on the efficacy of nurses replacing doctors prior to a patient receiving a diagnosis [38]. In a systematic review of CHW studies in the United States, Viswanathan and colleagues found mixed evidence on participant behaviour change and health outcomes [39]. Supervision and training is an important component for quality of care. Barber et al. found quality improvements at public health facilities in Indonesia that had at least one physician versus those that had none [16]. The Ministry of Health in Mozambique suspended training of non-physician clinicians providing antiretroviral therapy until the training program could be revised, because of poor quality of care results [40]. However, the particular type of supervision and training is sometimes difficult to measure and replicate in other settings.

The third finding is conceptual. When tasks have been shifted from traditional professional cadres (e.g. specialists, doctors or nurses) to new professional cadres, most studies compare the new cadre's productivity and patient outcomes to the traditional cadre's. The parallel comparison occurs between higher- and lower-skilled workers. However, the appropriate comparison is between the results from the care received by the new cadre and the results from the care the patient would have received--if any care at all--had the new cadre not been available. Verteuil articulated this point well in his response to Kruk et al.'s Mozambique study: "An appropriate comparator to *tecnicos de cirurgia *would be a 'do nothing' comparator as opposed to using formally trained surgeons....a more realistic alternative for patients treated by *tecnicos de cirurgia *would be no formal treatment at all, which would, it is presumed, result in far worse outcomes for the patients" [28] (p. 1260). Additionally, the opportunity cost of task shifting needs be incorporated into an evaluation, because a cadre that has shifted tasks will no longer be able to perform its original tasks.

The use of cost effectiveness analysis helps ensure appropriate comparisons are made. For example, Hounton et al. found newborn case fatality rates after a caesarean section in Burkina Faso were highest among those performed by clinical officers (198 per 1000) versus general practitioners (125 per 1000) and versus obstetricians (99 per 1000) [41]. By calculating the incremental cost effectiveness ratio, they found that the cost per avoided newborn fatality was only $200 when 1000 caesarean deliveries were performed by a general practitioner instead of a clinical officer, but the cost per avoided newborn fatality increased to $11 757 when 1000 caesarean deliveries were performed by an obstetrician versus a general practitioner (dollars expressed in 2006 United States dollars).

To generalize potential savings from task shifting, Scheffler et al. use simulations to illustrate how skill mix changes can mitigate overall wage bill gaps in sub-Saharan Africa in 2015 [42]. They estimate that 31 sub-Saharan Africa countries will experience needs-based health workforce shortages in 2015, and estimate the annual wage bill required to eliminate these shortages to be approximately $2.6 billion (2007 U.S. dollars). Their simulations show this wage bill could be reduced, for example, by between 2% and 5% by increasing the needed nurse-plus-midwife-to-doctor ratio by 50%, assuming a nurse or midwife is between 0.7 and 0.9 as productive as a doctor. Fulton and Scheffler extend this simulation to include CHWs (as discussed in Section 2 of this article), and Babigumira and colleagues used a time-motion survey of CHWs and other workforce cadres to estimate savings from task shifting [8,43]. The simulations provide a framework for policy makers to assess their own health workforce mix in the context of resource constraints.

## Discussion

### Proposed research agenda

Based on these three key findings, the research agenda should include studies that evaluate the impact of skill mix changes, particularly task shifting, on productive efficiency. It is important that the studies use an appropriate research design to estimate the effect of skill mix changes on patient health outcomes, quality of care, and costs. The particular areas of study should be based on local conditions, driven by the burden of disease and the areas where task shifting could have the most benefit, such as HIV/AIDS, malaria, tuberculosis, maternal health including obstetric surgery, children's health, and chronic conditions (e.g. see Lopez et al. [44]). These areas closely align with the health-related United Nations Millennium Development Goals (MDG). The studies should seek to determine whether health care services of a given quality are being produced at the lowest cost. For example, Walker and Jan critically review cost-effectiveness studies involving community health workers [45].

The role of new technologies, including e-health and telemedicine, needs to be considered (e.g. see Chandrasekha & Ghosh [46]). Information and communication technology (ICT) can influence the geographical need and training requirements for health workers. For example, in Kenya, community-based antiretroviral therapy care was augmented with pre-programmed personal digital assistants with decision support [26]. For complicated HIV/AIDS cases in Zambia, health workers consulted HIV clinicians in the United States, Canada, and South Africa via the internet [47]. Technology can profoundly modify the skills required, for example, by shifting the need for invasive and life-threatening surgical skills in favour of medical treatment or non-invasive procedures that can be performed by technicians.

A randomized trial is the best research design to estimate the causal effect of a particular policy intervention--in this case, a skill mix change--on a particular outcome. However, randomized controlled trials tend to lack external validity, because the study is testing a specific intervention within a specific context, defined by factors such as the health system, payment scheme, workforce training, and management culture. Therefore, it is important to not only estimate the main effect of task shifting policy, but to also estimate how the effect is influenced by contextual factors. Because of ethical, logistical and political economy issues, randomized controlled trials are sometimes not feasible, so quasi-experimental designs need to be utilized, but they carry the same external validity concerns. Ideally, multi-country studies should be conducted using a similarly rigorous experimental design. This would be a priority area for the international community to support.

Case studies, including the comparison of different health care providers, are another important research design. For example, a provider group or facility that produces high-quality health care at low costs can be studied to better understand the management, supervision, skill mix, training, incentives, and processes that produce these results. These findings can also inform the skill mix interventions that should be tested with a randomized controlled trial. More emphasis needs to be given to these contextual and enabling factors that determine whether task shifting will be effective (e.g. for community health workers, see Lehmann and Sanders; for community health workers providing HIV services, see Celletti et al. and Hermann et al.) [48-50]. These contextual factors include patients' acceptance of the cadre's new role, such as a community health worker [50].

Two cases studies from Pakistan and Ethiopia are discussed to illustrate the importance of contextual and enabling factors. A recent review of the Pakistan Lady Health Worker program suggests contextual factors are important in determining the success or failure of a skill mix policy change [51]. There was high-level political support for this program--at the level of prime minister. The lady health workers had to be residents of the community in which they work. Each lady health worker was attached to a government health facility from which she received training, a small allowance, and medical supplies. Candidates had to be recommended by the community and meet a set of criteria, including having a minimum of eight years of education. Further study is needed to determine which of these factors were most important relative to their cost in enabling the program to achieve better health outcomes as compared to the control population.

Similarly, the community-based health extension workers (HEW) within Ethiopia's Health Extension Program offer insight into the potential importance of contextual factors, particularly the use of HEWs in remote areas [18]. Some of the factors identified include leadership and training (e.g. mentoring, continuing education, supervision, monitoring), workplace infrastructure (e.g. buildings, equipment, supplies, reference material) and living conditions (e.g. housing, transportation, relationship with community). Given that the Health Extension Program has a limited budget, it is important for future studies to identify which factors are most important relative to their cost.

### Study limitations

This article includes four limitations that warrant discussion. First, the literature review focused on studies published in 2006 or later, but included some studies with strong evidence prior to 2006. While the review may have omitted particular studies, we do not think their inclusion would change the main findings of this article, given the substantial evidence presented by the included studies. Second, there is a bias for investigators to submit, and editors to publish, studies based on the direction or strength of the findings, which is known as publication bias [52]. Within published studies, there is a bias to selectively report these same types of outcomes, known as outcome reporting bias [53]. It is difficult to estimate the effect of this potential bias, but it is likely be present given its pervasiveness. However, its effect is somewhat mitigated in studies involving task shifting, where a finding of no significant differences (e.g. on patient quality of care measures or outcomes) between workforce cadres is an important finding that will likely be published. Third, many of the included studies involved small sample sizes, limiting their ability to detect differences between workforce cadres. Larger-sample studies in the future will add important information. Fourth, countries have different entry and education requirements for health workers (e.g. non-physician clinicians) and the included studies used different training interventions for cadres [21]. Comparisons across countries and studies need to control for these differences.

### Information gaps

Recent evidence in developing countries shows that the major information gaps in health policy are not on 'what to do' but rather on implementation - 'how to do it' [54]. The 'how to do it' depends on contextual factors, and WHO developed a series of research questions to be asked, including the following [55]:

• What are the country-specific factors that will guide decision-making in the implementation of task shifting?

• What preconditions must be met for the safe, efficient and effective implementation of task shifting?

• How can countries create enabling conditions for task shifting through an appropriate regulatory framework?

• What measures must be taken to ensure quality of care under the task shifting approach?

• How can task shifting be implemented in a way that is sustainable [both politically and fiscally]?

Some of these questions, however, suggest that there is strong evidence that the current skill mix and task allocation are the most productively efficient, implying that task shifting represents a risk. However, in many cases, the evidence either does not exist or is based on weak research designs. Current task allocation is often influenced by tradition and the political power of health worker cadres. In many low income countries, task shifting may be an essential strategy to improve service delivery, because of health worker shortages, low productivity, and low quality of care. Therefore, some other questions could be added to the above list, such as:

• What is the evidence that shows the current skill mix is productively efficient?

• Is the current skill mix responding to the country's needs?

• What skill mix is needed to improve the country's health indicators?

• Which skill profiles provide more productively efficient care delivery?

• What are the constraints to introduce flexibility into education and training policies to adjust the skill mix and each cadre's activities and tasks to evolving needs and technology?

• What informal task shifting is occurring outside scope of practice regulations?

While studies can identify the primary contextual factors that influence which skill mix is most productively efficient in a particular setting, there are too numerous combinations of factors to test them all. Therefore, it is important that the health care system include the necessary incentives for health care administrators to use the most productively efficient skill mix in their local setting.

## Conclusion

In summary, by providing health care services at the productively efficient skill mix--the mix that produces the maximum number of health care services at a given quality and cost--more health care services are going to be accessible and affordable to populations seeking care. Task shifting is a policy option that should be considered to help achieve productive efficiency and provide access to services that otherwise might not be available. A more productively efficient skill mix will partially dampen the effect of health workforce needs-based shortages and better enable countries to meet the health-related United Nations Millennium Development Goals.

## Competing interests

The authors declare that they have no competing interests.

## Authors' contributions

BF participated in the study concept and design, acquisition and interpretation of studies, and drafting the manuscript. RS participated in the study concept and design, interpretation of the studies, and critically revising the manuscript for important intellectual content. SS participated in the acquisition and interpretation of the studies and drafting the manuscript. EA, AS, and MV participated in the study concept and design, and drafting the manuscript. All authors read and approved the final manuscript.

## Supplementary Material

Additional file 1 **Studies analyzed **[2,5,16,20-23,25-30,32,36-38,40-42,56-66]. The details of the 31 studies that we analyzed are included in Table 1 within Additional file 1. Click here for file
