# Supplementary material for: Health workforce skill mix and task shifting in low income countries: a review of recent evidence
Source: Hum Resour Health. 2011 Jan 11;9:1. doi: 10.1186/1478-4491-9-1 (PMC3027093; doi:10.1186/1478-4491-9-1)
Supplement: Additional file 1 — Studies analyzed [2,5,16,20-23,25-30,32,36-38,40-42,56-66]. The details of the 31 studies that we analyzed are included in Table 1 within Additional file 1. [file 1478-4491-9-1-S1.DOC]

# Additional file 1 - Studies analyzed

The details of the 31 studies that we analyzed are included in Table 1. The studies are grouped by the following topics: health care (various types), primary care, maternal care and obstetric surgery, HIV/AIDS, children, and mental health. Each row in the table represents a study, and the columns include the elements we used to describe and evaluate the studies.

## Table 1 - Key Elements of the 31 Studies that Were Analyzed

| **No.** | **Type of health care** | **Authors and public-ation year** | **Research questions** | **Population studied** | **Study design** | **Analytic method** | **Key results and comments** |
| --- | --- | --- | --- | --- | --- | --- | --- |
| 1 | Health care (various types; hospi-tals) | Herbert-son, Blundell, & Bowman, 2007 [56] | Are trained clinical support workers able to reduce junior doctors’ workloads in teaching hospitals while maintaining the same quality? | United Kingdom, 2000-2001, Nottingham City Hospital;  n = 1264 cannulations;  n = 1513 venepunctures;  n =5 clinical support workers | Multi-group comparison (non-random assignment) | Quantitative: descriptive statistics | The study found that clinical support workers were able to significantly reduce the number of cannulations and venepunctures performed by junior doctors. The clinical support workers had a cannulation success rate of 94% and a venepuncture success rate of 95%. Authors state that these rates did not compromise patient care. One limitation is that only five clinical support workers were included in the study. |
| 2 | Health care (various types) | Mullan and Frehywot, 2007 [21] | In what countries are non-physician clinicians practicing and what are their roles? | 47 sub-Saharan Africa countries, 2007, key informant interviews | Case study | Qualitative | The study reports that non-physician clinicians (NPCs) exist in 25 of 47 sub-Saharan Africa countries, but note the lack of comprehensive data on NPCs. NPC definitions vary across countries with respect to pre-service education, training, and tasks. They find that NPCs provide a wide array of clinical services, including specialty activities such as caesarean section, ophthalmology, and anaesthesia. NPCs are less costly and take less time to train than physicians. Their training is highly practical and localized to the specific needs of the community in which they serve, but there is a lack of standardization of training and roles within and across countries. |
| 3 | Health care (various types) | Buchan and Dal Poz, 2002 [25] | What is the evidence base and limitations of skill mix studies? | Global, 1986-2000; meta-analyses, single site, and large-scale data surveys, 41 references | Literature review | Structured | The literature review found that the literature on skill mix was primarily descriptive in nature and had methodological weaknesses. The majority of the studies were USA-focused, which may not applicable to other contexts. The review found that the increased use of less-qualified health workers will not necessarily be effective in all contexts; however, there is evidence that increasing the scope of nurses and midwives may be beneficial in some contexts. |
| 4 | Health care (various types) | Dovlo, 2004 [2] | What is the evidence base on substitute health workers with respect to education, regulation, scope of practice,  and cost-effectiveness? | WHO documentation, ministry of health documents and internet search focused on sub-Saharan Africa, 1987-2003, 22 references | Literature review | Structured | The literature review found that substitute health workers were cost effective in African health systems. Studies found quality is similar between doctors and clinical officers. Retention of substitute health workers is higher than professional workers, particularly in rural areas. The review concludes that in order to make the most effective use of substitute health workers, it is important to understand the various roles and conditions under which each cadre is most effective. Professional scopes of practice will need to change to permit effective substitution among cadres. |
| 5 | Health care (various types) | Hongoro and McPake, 2004 [5] | How can skill mix changes reduce the needs-based shortage in human resources for health? | Low-income countries, 1978-2004 | Descriptive study | Qualitative | The study found that the capacity to train doctors and nurses in low-income countries is highly constrained. The increased use of auxiliary health workers, such as nurse aides, medical assistants, and clinical officers, has been successful. Moreover, the demand for these workers in high-income countries is less than for doctors and nurses. More research needs to be conducted in order to better understand effectiveness and document the roles of auxiliary health workers. |
| 6 | Health care (various types) | Hooker, 2006 [20] | How many physician assistants (PA) and nurse practitioners (NP) are in the United States, and what are their roles? | United States, 2006, nurse practitioner and physician assistants | Case study | Qualitative | The study found that as of 2006, 110 000 physician assistants and nurse practitioners comprised one-sixth of the United States of America’s medical workforce, with an additional 11 200 graduating each year. They can provide almost 90% of the services that a primary care physician can provide. PAs and NPs are employed by over one-quarter of all group practices, and are major sources of patient access in rural areas and in large health maintenance organizations. |
| 7 | Health care (various types) | Scheffler et al., 2009 [42] | What is estimated health workforce needs-based shortage in 2015, and how would skill mix changes reduce the wage bill shortage? | 39 sub-Saharan African countries, 2015; physicians, nurses, and midwives | Forecast | Quantitative: multi-variate regression and simulations | The study estimates that 31 of the 39 sub-Saharan African countries analyzed will experience a needs-based shortage of doctors, nurses and midwives in 2015. The estimated annual wage bill required to eliminate these shortages is approximately $2.6 billion (2007 U.S. dollars). Their simulations show this wage bill could be reduced, for example, by between 2% and 5% from increasing the needed nurse-plus-midwife-to-doctor ratio by 50%, assuming a nurse or midwife is 0.7 to 0.9 as productive as a doctor. |
| 8 | Primary care | Kinner-sley et al., 2000 [57] | What are the differences between care from nurse practitioners and general  practitioners for patients seeking ‘same day’  consultations in primary care? | Wales and England, 1999;  10 general practices in south Wales and south  west England. 1368 patients requesting same day  consultations | Randomized controlled trial. Patients seeking same-day consultations were randomly assigned to a nurse practitioner (NP) or general practitioner (GP), sometimes in blocks. | Quantitative | The study found that most patients reported at two weeks after their consultation that their symptoms had improved and their concerns were reduced, but there was no statistical difference between patients who were treated by a GP versus an NP. For children, mean satisfaction level was statistically higher for NP (80.4) versus GP (75.6), based on a 100-point scale. For adults, mean satisfaction level was statistically higher for NPs versus GPs in 3 of 10 practices; no statistical differences were found in other practices. Consultation times were shorter for GPs, even after accounting for time taken by NPs to get prescriptions signed. In the 10 practices, the consultation time GP:NP ratio ranged from 0.57 to 0.92, eight of which were statistically different from 1. Patients were significantly more likely to receive particular information from NPs versus GPs, including cause of illness (81% vs. 72%), how to relieve symptoms (86% vs. 68%), and what to do if the problem persists (93% vs. 88%). |
| 9 | Primary care | Gary et al., 2009 [58] | What are clinical characteristic differences and emergency room utilization differences between Type 2 diabetes mellitus patients assigned to minimal intervention care versus minimal intervention care plus individ-ualized,  culturally tailored care provided by a nurse case manager (NCM) and a community health worker (CHW)? | United States, 2001-2003;  managed care organization in Baltimore, Maryland; n= 542 African Americans with Type 2 diabetes mellitus | Randomized controlled trial. Patients randomly assigned to either minimal intervention care or minimal intervention care plus individualized,  culturally tailored care provided by a NCM and a CHW (i.e., intensive intervention group). | Quantitative, multi-variate regression | The study found that at 24 months, patients who had more visits with a CHW and NCM approached having a statistically significant decline in HbA1c levels (−0.43%) as compared with the minimal group (p = 0.12). The study also found that at 24 months, patients in the intensive intervention group were 23% less likely to have emergency room visits compared with the minimal intervention group. |
| 10 | Primary Care | Barber et al., 2007 [16] | What are the contributions of physicians, nurses, and midwives to the quality of primary health care? | Indonesia; 1993 and 1997; nationally representative health facilities (n = 992 in 1993; n=915 in 1997) | Quasi-experimental | Quantitative: multi-variate regression | The study found that quality of care depends on the availability, type and number of health workers. There was significant evidence that quality of care depends on physician presence at a facility. For example, prenatal care quality was 4.5 percentage points higher in a facility with one versus zero physicians, and 6.3 percentage points higher in a facility with two versus zero physicians. The study found larger impacts on improvements in curative care when an additional nurse is added as compared to an additional physician or midwife. |
| 11 | Primary care | Lewin et al., 2010 [23] | What is the effect of using lay health workers (LHW; e.g., community health workers) on primary care, particularly maternal and child health as well as infectious disease care? | Global review, 1975-2010,  n = 82 studies, including 55 studies in six high-income countries (many of these focused on low-income and minority populations), 12 studies in eight middle-income countries, and 15 studies in ten low-income countries | Literature review of randomized controlled trials | Systematic, meta-analysis | The review finds moderate-quality evidence that LHWs promote immunization uptake and breastfeeding and improve tuberculosis treatment outcomes, as compared to usual care. Low-quality evidence was found for LHWs reducing child morbidity and child/neonatal mortality and increasing the likelihood of seeking care for childhood illness, as compared to usual care.  Note for this review, low-quality evidence means that further research is very likely to have an important impact on the estimated effect, because, for example, the estimate did not have a *p-value* less than 0.05, although it was typically less than 0.10, and because heterogeneous effects were found across the studies. Moderate-quality evidence means that further research is likely to have an important impact on the estimated effect, while high-quality evidence means that further research is very unlikely to have an important impact. |
| 12 | Primary care (and hosp-itals) | Buchan and Calman, 2005 [38] | What are the implications of skill-mix changes between physicians and nurses in primary care and hospitals? | OECD countries, with 16 responses to a survey; literature review (139 references); U.S. and UK (case study); 1990 – 2004 | Literature review, case study | Unstructured, qualitative | The study found that the evidence regarding the effectiveness of replacing doctors with nurses was mixed in the USA, U.K. and Australia. Many studies examined the possible benefits of replacing doctors with nurses once a diagnosis had occurred; however, many questions remained on the efficacy of nurses when a diagnosis has not been reached. Of the 16 OECD countries that responded to the OECD survey, 8 countries reported using nurses in advanced practice, with 3 additional countries undergoing pilots in this area. In the USA. and UK, the key drivers of the increased use of nurses in advanced roles included value (cost savings), insufficient doctors, and the introduction of new treatments. The study noted that there is more support for the increased use of nurses in the USA as compared to the UK. |
| 13 | Maternal care and obstetric surgery | Kruk et al., 2007 [28] | What are the cost differences between surgically trained assistant medical officers and surgical specialist physicians to perform major obstetric surgeries in Mozambique? | Mozambique, 2002, 47 specialist physicians (5264 major obstetric surgeries) and 53 assistant medical officers (6914 major obstetric surgeries) | Multi-group comparison (non-random assignment) | Quantitative: descriptive statistics | The study found that the cost per major obstetric surgery (caesarean sections, obstetric hysterectomies, and laparotomies for ectopic pregnancy) is approximately one-quarter for assistant medical officers ($38.90) as compared to surgical specialist physicians ($144.10). These costs include training and wages over a 30-year career. Costs are reported in 2006 United States dollars. One limitation is that the non-surgical tasks were not included. |
| 14 | Maternal care and obstetric surgery | Pereira et al., 2007 [59] | What shares of major obstetric surgeries in Mozambique are done by surgically trained assistant medical officers versus surgical specialist physicians, and where do these cadres work? | Mozambique, 12 178 major surgical obstetric operations in 2002; 59 medical officers and 34 surgically trained assistant medical officers | Multi-group comparison (non-random assignment) | Quantitative: descriptive statistics | The study found that assistant medical officers performed 57% of all major obstetric surgical interventions in Mozambique, including 92% of the interventions in district (rural) hospitals. After seven years, no medical officers initially assigned to district (rural) hospitals remained there, while 88% of assistant medical officer graduates remained there. |
| 15 | Maternal care and obstetric surgery | Hounton et al., 2009 [41] | What is the cost effectiveness of cesarean sections provided by clinical officers, general practitioners, or obstetricians? | Burkina Faso, 2004-2005; 2305 cesarean sections | Multi-group comparison (non-random assignment) | Quantitative: descriptive statistics | The study found newborn case fatality rates after a caesarean section in Burkina Faso were highest among those performed by clinical officers (198 per 1000) versus general practitioners (125 per 1000) versus obstetricians (99 per 1000). Based on the incremental cost effectiveness ratio, the cost per avoided newborn fatality was only $200 when 1000 caesarean deliveries were performed by a general practitioner versus a clinical officer, but the cost per avoided newborn fatality increased to $11 757 when 1000 caesarean deliveries were performed by an obstetrician versus a general practitioner (dollars expressed in 2006 United States dollars). |
| 16 | Maternal care and obstetric surgery | Chilopora et al., 2007 [29] | What were patient outcome differences between patients receiving obstetric surgery from clinical officers versus medical officers? | Malawi, 2005; n=2131 obstetric surgeries | Multi-group comparison (non-random assignment) | Quantitative: descriptive statistics  (chi-square test) | The study did not find post-operative differences between patients receiving obstetric surgery from clinical officers versus medical officers, for the occurrence of pyrexia, wound infection or dehiscence, re-operation need, neonatal outcome, or maternal death. |
| 17 | Maternal care and obstetric surgery | McCord et al., 2009 [60] | What were patient outcome and quality of care differences between patients receiving obstetric surgery from assistant medical officers versus medical officers? | Tanzania, 2006; mothers who had major surgery by assistant medical officer (n=945) and by medical officer (n=142). | Multi-group comparison (non-random assignment) | Quantitative, mostly compared means | The study did not find significant differences between assistant medical officers and medical officers regarding patient outcomes and quality of care. Patient outcomes included maternal death, perinatal death, and major post-operative complications. Quality of care measures included whether surgery was performed without an absolute maternal indication or clear foetal indication; delay of surgery by more than three hours; and absence of a blood transfusion when needed. One limitation was that a multivariate regression model was not estimated to account for maternal and foetal risk indicators simultaneously with the hospital’s attributes, likely because of the relatively small sample size of surgeries performed by medical officers. |
| 18 | Maternal care and obstetric surgery | De Brouwere et al., 2009 [61] | What was the effect of task shifting on the numbers and rates of major obstetrical surgical interventions as well as its effect on maternal and perinatal outcomes? | Senegal, 2001-2006. n=3 districts. Task shifting included training non-specialists (general practitioners, anaesthetists, and surgical assistants) in emergency obstetric surgery. | Case study, hospital records reviewed; key informant interviews | Quantitative | The study found that caesarean-section rates increased in district hospitals with a functioning surgical team, with positive outcomes for newborns as well. The district of Bakel had the greatest increase in intervention rate, a 2.6-fold increase between 2001 and 2006. However, across the districts studied, varying availability of surgical teams jeopardized these positive effects, and the number of surgical teams was never sufficient to meet the need. |
| 19 | HIV/ AIDS | Selke et al., 2010 [26] | What were HIV/AIDS patient outcome differences between those treated with clinic-based care versus community-based care delivered by people living with HIV/AIDS who had pre-programmed personal digital assistants with decision support? | Kenya, 2006-2008, patients in clinic-based care (n=112), patients in community-based care (n=96) | Randomized controlled trial. Randomization unit was at the sub-location level within the Kosirai Division. Clinic-based care included monthly clinic visits, while community-based care included monthly community-based visits plus one visit every three months to a clinic. | Quantitative, multi-variate regression | The study did not find significant clinical outcome differences after one year between patients treated in the clinic versus the community. The outcomes included detectable viral load, mean CD4 count, decline in Karnofsky score, change in ART regimen, new opportunistic infection, and pregnancy rate. One noted limitation is the study’s limited power to detect differences, given the small sample size. During the one-year study period, the community-based care group had fewer visits per patient (6.2) than the clinical-based care group (12.4). |
| 20 | HIV/ AIDS | Sanne et al., 2010 [62] | What were HIV-infected patient outcome differences between those assigned to nurse- versus doctor-monitored anti-retroviral therapy (ART) care? | South Africa, 2005-2009, two primary care sites in Cape Town and Johannesburg; n = 404 HIV patients assigned to nurse-monitored ART care; n= 408 HIV patients assigned to doctor-monitored ART care | Randomized controlled trial: patients were randomly assigned nurse- or doctor-monitored ART care | Quantitative, regression analyses | The study found that after a median follow-up of 120 weeks that patients in the nurse- versus doctor-led care groups had similar outcomes: deaths (10 vs. 11), virological failures (44 vs. 39), toxicity failures (68 vs 66), and programme losses (70 vs. 63). Results support feasibility of appropriately trained nurses to monitor ART care. |
| 21 | HIV/ AIDS | Vasan et al., 2009 [27] | What is the degree of agreement between physicians and non-physicians on decision to start antiretroviral therapy (ART) in Uganda? | Uganda, 2006; 254 patients seen by a nurse and a physician; additional 267 patients seen by a clinical officer and a physician; locations included 12 government ART sites | Randomized controlled trial: patients were first randomly assigned to be seen by a nurse or clinical officer; patients were then seen by a physician | Quantitative, Kappa analysis | The study found that nurses and clinical officers both had considerable strength of agreement with physicians, particularly with final ART recommendation, WHO clinical stage assignment, and tuberculosis status assessment. Clinical officers and physicians had more agreement (weighted Kappa = 0.76) on final ART recommendation than the degree of agreement between nurses and physicians (weighted Kappa = 0.62). On the other hand, nurses and physicians had more agreement on WHO clinical stage than the degree of agreement between clinical officers and physicians. |
| 22 | HIV/ AIDS | Brent-linger et al., 2010[40] | How well did non-physician clinicians (NPC) perform on antiretroviral therapy (ART) clinical practice quality as compared to norms they were taught in their two-week course, as determined by two clinical observers? | Mozambique, 2007; public sector health facilities; n = 127 patients; n = 44 non-physician clinicians | Multi-group comparison (non-random assignment), where comparison group is a norm | Quantitative: descriptive statistics | The study found that the NPCs’ agreement rates with the clinical observers were as follows: staging (37.6%),  co-trimoxazole management (71.6%), ART  management (75.5%), ADR management (69.7%), and diagnosis of opportunistic infections and other infectious  diseases (49.1%). These low agreement rates caused the Ministry of Health to suspend training until the ART training program could be revised. |
| 23 | HIV/ AIDS | Wools-Kalous-tian et al., 2009 [63] | How well did community care coordinators (CCC) with personal digital assistants (PDA) perform on patient monitoring and antiretroviral dispensing tasks for HIV patients? | Kenya, Kosirai Division, 2006-2008; n = 133 and n = 88 patient evaluations in years 1 and 2, respectively; n = 8 CCCs | Multi-group comparison (non-random assignment), where comparison group is a norm | Quantitative: descriptive statistics | The study found that the CCCs performed well. On the patient evaluations, they received superior evaluations at the following rates for year 1 and year 2, respectively: summary score (88.7%, 94.3%), obtaining vital signs (90.8%, 100%), taking histories (91.9%, 98.8%), using the PDA (90.6%, 100%), making clinical judgments (83.1%, 98.8%), displaying humanistic qualities (86.4%, 95.5%), and interacting with staff (89.1%, 92%). |
| 24 | HIV/ AIDS | Shum-busho et al., 2009 [64] | How well did nurse-centred antiretroviral therapy (ART) compare with national guidelines for ART eligibility and prescription, and what were the key patient outcomes? | Rwanda, 2005-2008, HIV/AIDS patients in rural health centers (n=1076) | Case study, retrospective examination of patient records | Quantitative, descriptive statistics | The study found that nurses achieved high compliance with national guidelines for ART eligibility and prescription, and had excellent patient outcomes. Of the 622 patients determined ineligible for treatment, none had been started on treatment. Of the 451 patients determined eligible for ART, 435 had started treatment; the remaining 16 had not started treatment for various reasons, none attributed to the nurse. All prescriptions were consistent with national guidelines, except one patient was given efavirenz without prior exclusion of pregnancy. Of the 435 patients initiating ART, 90% were alive at assessment. One limitation was the lack of an explicit comparison group of health workers. |
| 25 | HIV/ AIDS | Callaghan et al., 2010 [22] | What is the effect of task shifting in HIV treatment on patient access, cost, and quality of care? | sub-Saharan Africa, searched databases from inception to May 2009 (n=25 original studies), 80 references | Literature review | Structured | The literature review found that task shifting effectively addresses health worker shortages in HIV treatment and care, because it provides high-quality care to more patients than a physician-centred model. Task shifting is cost effective, but may not be cost saving, because a greater number of people will be able to access services. Noted challenges include training, support and integration of staff into new roles, and compliance with regulatory bodies. |
| 26 | HIV/ AIDS | Zachariah et al., 2009 [36] | What are the primary opportunities and challenges for task shifting in ART in sub-Saharan Africa? | Malawi, South Africa and Lesotho; 2004-2007, National ART scale up plan (Medecins Sans Frontieres), 57 references | Case study; literature review | Qualitative; unstructured | The study highlights both the opportunities and challenges presented by task shifting for ART. Opportunities include improving the workforce skills mix and health-system efficiency; enhancing the role of the community; cost advantages; and reducing attrition and international ‘brain drain’. The challenges include maintaining quality and safety; addressing professional and institutional resistance; and sustaining motivation and performance. |
| 27 | HIV/ AIDS | McCourt and Awases, 2007 [65] | How has Namibia managed its health workforce, focusing on skill mix/ hierarchy, employee selection, performance appraisal, and training? | Namibia; 2005, 22 key informant interviews | Case study | Qualitative | The study concludes that while a general scaling up is needed, a focus on increasing number of low- and middle-levels cadres of health workers will create a more efficient allocation of resources. The study cites the use of job analysis and competence development to identify the precise skills that the new treatments will require, and to provide them to trainees at the lowest possible level, equivalent to Namibia's enrolled nurses and community counsellors. Additional higher-level cadres will need management training to supervise these workers. |
| 28 | HIV/ AIDS | Kober and Van Damme, 2004 [66] | What are the primary constraints and solutions to scale up anti-retroviral treatment in Africa? | Malawi, Mozambique, Swaziland, and South Africa, January 2004; interviews with country officials | Case studies | Qualitative | The study found that the primary barrier to scaling up antiretroviral treatment was not a lack of financial resources, but rather a scarcity of health workers to deliver the treatment. In order to meet these needs, countries are scaling up medical schools, improving secondary schools to improve the number of applicants, and using lower-skilled cadres. |
| 29 | Children | Rowe et al., 2007 [37] | How well did community health workers diagnose and treat ill children in Siaya District of Kenya? | Kenya (Siaya District), 2001, 192 ill-child consultations performed by 114 CHWs | Multigroup comparison (non-random assignment) | Quantitative: descriptive statistics | The study found that CHWs performed 80% of all guideline-recommended procedures correctly, but only 58% of ill children were prescribed all potentially life-saving treatments. The CHWs were evaluated by physicians who were experts with CARE Management of Sick Child (MSC) Guidelines; therefore, physicians were the comparison group. |
| 30 | Children | Huicho et al., 2008 [30] | How does quality of care of children under age 5 differ among health workers (physicians, nurses, midwives, clinical officers) with different number of years of pre-service training, but who received in-service Integrated Management of Childhood Illness (IMCI) training? | Bangladesh (2003, n=272 children),  Brazil (2000, n=147 children), Uganda (2002, n=612 children), Tanzania (2000, n=231 children) | Multi-group comparison (non-random assignment). Within each country, health workers were classified into either shorter or longer duration training, based on the number of years of pre-service training they had received. | Quantitative, t-tests, chi-square tests, multi-variate regression | The study found that among the 12 comparisons (three outcomes by four countries), pre-service training was not found to be significant for nine of the comparisons. The three outcomes included an index of integrated assessment of children (continuous measure), children correctly classified/diagnosed (binary measure), and children correctly managed/treated (binary measure). However, in Tanzania, workers with longer duration pre-service training had better assessment index scores than those with shorter duration pre-service training: 0.94 vs. 0.88, adjusted difference equal to 0.06. In contrast, in Uganda and Brazil, workers with longer duration pre-service training had worse management/treatment results than those with shorter duration pre-service training: Uganda (23.1% vs. 32.6%, adjusted odds ratio equal to 0.59) and Brazil (57.8% vs. 83.7%, adjusted odds ratio equal to 0.11). |
| 31 | Mental health | Rahman et al., 2008 [32] | What is the change in depression prevalence among depressed, pregnant women treated by lady health workers routinely trained versus specially trained using the Thinking Healthy Programme (cognitive behaviour therapy techniques)? | Pakistan, 2005-2006, women aged 16-45 in third trimester of pregnancy who had clinical depression, based on the Diagnostic and Statistical Manual of Mental Disorders, Fourth Edition (DSM-IV). Women were visited by a lady health worker during and for 10 months following pregnancy. Control group (n=463); treatment group (n=440). Randomization occurred at Union Council level (population of 15 000 to 20 000). Forty Union Councils participated. | Randomized controlled trial | Quantitative, multi-variate regression | The study found that women who were clinically depressed during pregnancy had better mental health outcomes if they were visited by lady health workers trained with the Thinking Healthy Programme as compared to women in the control group. Specifically, women in the treatment group had lower depression prevalence (23%) than women in the control group (53%) at 6 months after giving birth. Similarly, women in the treatment group had lower depression prevalence (27%) than women in the control group (59%) at 12 months after giving birth. The results remained statistically different when independent variables were included in the model, such as mother’s baseline depression score, age, education, and socioeconomic status. |
